# γ-Aminobutyric Acid Intake Improves Psychological State and Performance in Esports: A Randomized, Placebo-Controlled, Double-Blind Crossover Study

**DOI:** 10.3390/nu17111870

**Published:** 2025-05-30

**Authors:** Yoshihiro Hara, Utano Nakamura, Keita Koga, Yusuke Yamashita, Youngil Kim, Goichi Hagiwara, Ryousuke Furukado

**Affiliations:** 1 R&D Division, Pharma Foods International Co., Ltd., 1-49, Goryo-Ohara, Nishikyo-ku, Kyoto 615-8245, Japan; y-hara@pharmafoods.co.jp (Y.H.);; 2 Faculty of Human Science, Department of Sport Science and Health, Kyushu Sangyo University, 2-3-1 Matsukadai, Higashi-ku, Fukuoka 813-8503, Japan; hagi-g@ip.kyusan-u.ac.jp; 3 Faculty of Engineering, Integrated System Engineering, Nishinippon Institute of Technology, 1-11, Aratsu, Miyako-gun, Fukuoka 800-0394, Japan

**Keywords:** γ-aminobutyric acid, GABA, esports, psychological state, performance

## Abstract

**Objective**: This study focused on the effect of γ-aminobutyric acid (GABA) intake on psychological state and game performance during esports gameplay. **Methods**: A randomized, double-blind, placebo-controlled crossover study was conducted with eight healthy male university students aged 20–24 years who regularly play esports. The participants ingested either 200 mg of GABA or a placebo prior to gameplay and then completed a standardized esports task using the Mobalytics Proving Ground™ (MPG), a perceptual-cognitive task within “League of Legends” designed to train and evaluate player performance. Subjective psychological states were assessed pre- and post-gameplay using the Profile of Mood States 2 (POMS2^®^) short version. Esports task performance was evaluated based on MPG scores. **Results**: GABA intake significantly reduced psychological confusion–bewilderment and fatigue in the POMS 2^®^ short version during esports gameplay. Furthermore, the game scores were significantly higher in the GABA group compared to the placebo group. **Conclusions**: These findings suggest that GABA intake may serve as a potential strategy to enhance both the mental state and performance of esports players.

## 1. Introduction

The global esports market is growing rapidly, with revenues exceeding USD 1 billion in 2019 and estimated to have reached USD 1.79 billion in 2022 [1]. The audience of professional esports was estimated to be approximately 589 million people worldwide in 2020 [2]. Furthermore, the International Olympic Committee considers esports an official Olympic event, and esports are attracting increasing attention worldwide [1].

Esports involve high-tension gameplay to achieve victory, and players are exposed to various stressors during competitions [3]. Similarly to traditional sports, esports performance depends on psychological states [4]. Studies on the relationship between esports and psychological states have increased in recent years [5], and effective methods are required to regulate the psychological states of esports players and enhance their performance.

Nutrients are now recognized as key contributors to performance in sports, including esports. For example, in physical sports, the ingestion of low-to-moderate doses of caffeinated coffee has been shown to improve lower-body muscular endurance and cognitive performance in caffeine-naive female athletes without increasing cardiovascular load [6]. Caffeine, when combined with L-theanine or guarana, may also improve accuracy and cognitive function in precision sports such as curling and modern pentathlon [7,8].

Recent studies have also shown that nutrients may affect esports performance. Caffeine intake has been reported to improve shooting performance and reaction time [9], while energy drink consumption has been shown to enhance aspects of cognitive performance, such as working memory, in elite “League of Legends” players [10]. Although several nutrients have been reported to affect esports performance, few can effectively improve the psychological state required for esports, which involves complex operations and high stress levels. Therefore, nutrients capable of supporting both psychological state and optimal performance are considered essential in the context of esports.

γ-Aminobutyric acid (GABA) is an amino acid that influences the psychological state. GABA is a major inhibitory neurotransmitter of the central nervous system in humans and is widely found in common foods such as vegetables, fruits, and grains [11]. GABA is biosynthesized by glutamate decarboxylase (GAD) from glutamate in the bodies of animals, plants, and microorganisms, including humans. Numerous studies have also confirmed the safety of GABA, with a no-observed-adverse-effect level (NOAEL) established at 2500 mg/kg body weight per day [12,13,14].

GABA intake reduces temporary mental stress and fatigue and improves cognitive function [15,16]. Yoto et al. [17] reported that GABA intake reduces the deterioration of subjective vitality that occurs when individuals are subjected to temporary mental stress, such as computational problems. These studies measured the effect of GABA intake using cognitive function tests and psychological state (mood) as outcomes; however, the effects of GABA intake on psychological states during esports gameplay and its impact on gaming performance remain unclear.

In the present study, we aimed to clarify the effect of GABA intake on psychological state and game performance in esports gameplay, which requires multiple operations simultaneously, using psychological evaluation indices.

## 2. Materials and Methods

### 2.1. Participants

Nine healthy male university students aged between 20 and 24 years participated in this experiment (age: 21.33 ± 1.22 years; height: 168.71 ± 7.38 cm; body weight: 60.89 ± 13.63 kg). All participants had experience playing “League of Legends” (LoL) for approximately 2 years (23.31 ± 11.81 months), with an average of 3.33 ± 1.25 h of gameplay per week and a frequency of 3.33 ± 1.63 times per week. Therefore, they were deemed suitable for participation in this study. The exclusion criteria were as follows: (a) taking medication or under medical treatment; (b) pregnant, lactating, or intending to become pregnant during the study period; (c) under exercise or dietetic therapy; (d) allergic to the test supplement; (e) current dependence or history of either medicine or alcohol dependence syndrome; (f) current or prior diagnosis of mental illness (depression) or sleep disturbance; (g) extremely irregular lifestyle; (h) on a night-shift or shift work pattern; (i) unbalanced diet; (j) current disease or history of brain disease, malignant tumor, immunological disease, diabetes, renal disease, cardiac disease, thyroid disease, adrenal disease, or other metabolic disease; (k) use of health foods, supplements, and medicines; (l) participation in other clinical studies within the past three months or intention to participate in other clinical studies during the current study; (m) considered as an unsuitable candidate by the physician.

### 2.2. Procedures

A randomized, double-blind crossover trial was conducted in this study. The sample size for this trial was determined on the basis of previous studies that examined GABA intake [18] and psychological status (mood state) in esports [19]. The participants were randomly assigned to either the test supplement or placebo group for the initial phase of the crossover trial using random numbers generated in Microsoft Excel (2022). The allocation sequence was concealed using opaque, sealed envelopes and was blinded to the participants, investigators, caregivers, and outcome assessors until the end of the study.

**Figure 1:**
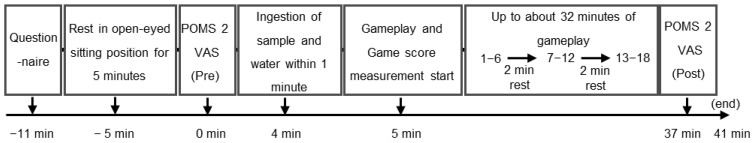
Study design and experimental protocol.

The participants participated in a crossover study with a one-week washout period. The participants were forbidden from eating or drinking anything other than water for two hours before the test. The details of the experimental procedure are shown in Figure 1. The participants entered the experimental room and completed questionnaires regarding their demographics (age, height, and weight) and gaming experience. They sat at rest with their eyes open for 5 min. After completing the POMS 2^®^ short version (Pre), the participants were instructed to drink GABA (or dextrin as a placebo) wrapped in a thin, tasteless film (oblate) with 30 mL of water within 1 min. Participants performed a 1 min Mobalytics Proving Ground™ (MPG) game, after which game scores were collected over a 30 s period. This session was repeated 18 times, with 2 min breaks introduced after the 6th and 12th sessions. During the 30 s score collection, the participants were instructed to sit quietly. Immediately after the 18th game, the POMS 2^®^ shortened version (Post) was administered.

### 2.3. Test Product

Pharma GABA^®^ (more than 95% purity of naturally fermented GABA, Pharma Foods International Co., Ltd., Kyoto, Japan) was used in this study. GABA (200 mg) was enveloped in a thin, tasteless film (oblate), whereas for the placebo, GABA was replaced with dextrin (Sandec^®^ ## 100, Dextrose Equivalent 10–13, Sanwa Starch Co., Ltd., Nara, Japan). The test supplement was prepared to be indistinguishable from the control in terms of appearance, texture, and smell to maintain blinding integrity.

### 2.4. Assessment of Psychological State (Mood State)

The Profile of Mood States Second Edition short version (POMS 2^®^ short version; Kaneko Shobo, Inc., Tokyo, Japan) was utilized to assess psychological state using its Japanese version. The POMS 2^®^ short version is a versatile index used to measure fatigue and relaxation in experimental studies and in the course of treatment of neurological disorders [20]. The POMS 2^®^ short version includes the following seven subscales: tension–anxiety (TA), depression–dejection (DD), anger–hostility (AH), vigor–activity (VA), fatigue–inertia (FI), confusion–bewilderment (CB), and friendliness (F). These subscales provide a comprehensive assessment of transient mood states across multiple dimensions. The total mood disorder (TMD) score (excluding subscale F) was calculated as follows:

TMD=(AH+CB+DD+FI+TA)−VA

The test consisted of 35 questions on mood states classified into seven scales. The examinee selected one of five levels (not at all: 0 to very often: 4) for each item. Raw scores were converted into T-scores based on normative data, allowing for standardized interpretation. T-scores (standardized T-scores) are transformed scores with a mean of 50 and a standard deviation of 10 and are used to compare individual performance to a normative population. Lower T-scores indicate better psychological conditions for AH, CB, DD, FI, TA, and TMD, whereas higher T-scores indicate better psychological conditions for VA and F.

### 2.5. Adopted Game and Mobalytics Proving Ground™ Assessment

A training game, called the Mobalytics Proving Ground™ (MPG; https://pg.mobalytics.gg/ (accessed on 27 February 2023)), was utilized to evaluate game performance in this study [21]. MPG serves as a perceptual–cognitive task within LoL, a leading example of the multiplayer online battle arena (MOBA) game genre, for training and evaluating game player performance [21]. Three game skills were measured using this online application: mechanics (indicating aim), background processing, and map awareness. Additionally, the total score was calculated using the scores from the mechanics, background processing, and map awareness tasks. Although the method for calculating the total score from the three individual tasks has not been disclosed by the game developers, the criteria for additions and deductions for each score were as follows. Mechanics was taken as the ability to manipulate the mouse in response to perceptual stimuli, which was evaluated using randomly displayed targets. Accurate clicks on the targets increased the points, whereas inaccurate clicks or failure to click before the targets disappear from the screen decreased the points. Background processing was evaluated using four randomly decreasing bars over several seconds each. The participants earned points by pressing one of the designated keys (Q, W, E, or R) on a computer keyboard when the corresponding bar on the computer screen changed from red to green. If the participants pressed the wrong key or missed the color change, the bar was locked for 5 s, and the participants lost points. Map awareness was assessed by monitoring the mini-map and navigating to avoid obstacles, using the F key to move right and the D key to move left on a computer keyboard.

### 2.6. Analysis

The participant demographics are presented as mean ± standard deviation (SD). The psychological state and game score values are presented as mean ± standard error (SE). The estimated marginal means (EMMs) and their 95% confidence intervals are presented in the text. For the analysis of the POMS 2^®^ short version, an analysis of covariance (ANCOVA) was performed with the pre-intake T-scores as covariates. The impact of the test supplement compared to the placebo on the game performance was evaluated using a mixed-effects model with intervention and sequence as fixed variables and participants as a random effect. Pairwise comparisons were conducted using the least significant difference (LSD) method. The significance level for this analysis was less than 5% (*p* < 0.05). IBM SPSS Statistics ver. 30 (IBM Corp., Armonk, NY, USA) was used to analyze the data.

## 3. Results

### 3.1. Participant Demographics and CONSORT Flow Diagram

**Figure 2:**
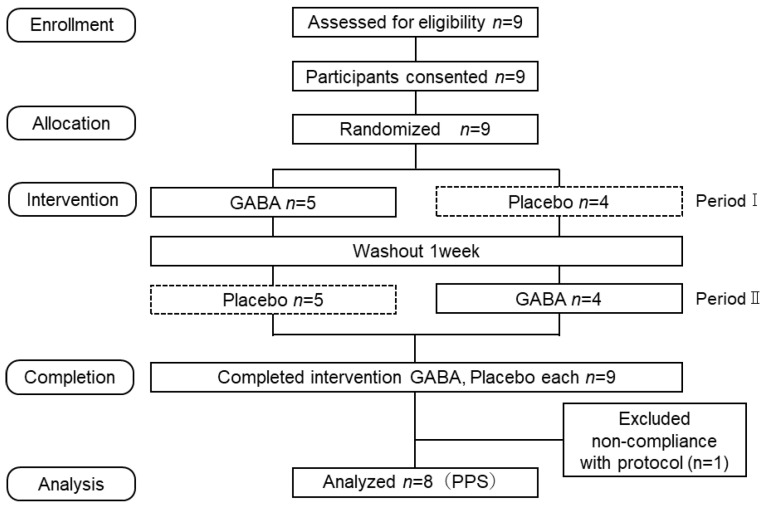
CONSORT flowchart of study participants.

A flowchart of the participant recruitment and analysis is shown in Figure 2. Eligible healthy volunteers provided informed consent to participate in the study and were randomly assigned to either the GABA-first (*n* = 5) or placebo-first (*n* = 4) groups. A total of nine participants completed the study. One participant was excluded due to the intake of hay fever medications during the study period. Consequently, eight participants were included in the final analysis. As of March 2024, based on the matchmaking rate (MMR) on the Japan server, four participants were unranked, while the remaining four were distributed across different tiers: one in bronze, one in silver, and two in platinum.

### 3.2. Effects of GABA Intake on the Assessment of Psychological State

**Figure 3:**
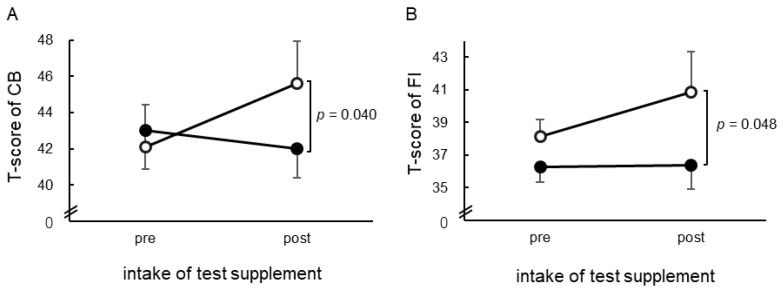
Effects of GABA intake on psychological state during the examination: T-scores for (**A**) confusion–bewilderment (CB) and (**B**) fatigue–inertia (FI) from the POMS 2^®^ short version are shown. Closed circles (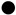) indicate the GABA group, while open circles (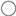) indicate the placebo group. Before (Pre) refers to the state prior to GABA or placebo intake, while after (Post) refers to the state following the GABA or placebo intake. The results indicate the mean ± standard error (SE). Significant differences were examined using ANCOVA (*n* = 8).

The results for CB and FI (T-scores) from the POMS 2^®^ short version are shown in Figure 3. After esports gameplay, the GABA group (42.00, 95% CI: 39.63, 44.36) exhibited significantly lower CB scores compared to the placebo group (45.63, 95% CI: 43.26, 48.00) (*p* = 0.040) (Figure 3A). Additionally, the GABA group (35.77, 95% CI: 32.33, 39.21) exhibited significantly lower FI scores compared to the placebo group (41.48, 95% CI: 38.04, 44.92) after the intervention (*p* = 0.048) (Figure 3B). No significant differences were observed in the other parameters. The tests for carryover and timing effects showed no significant differences for any item (Table S1).

### 3.3. Effect of GABA Intake on Game Score

**Figure 4:**
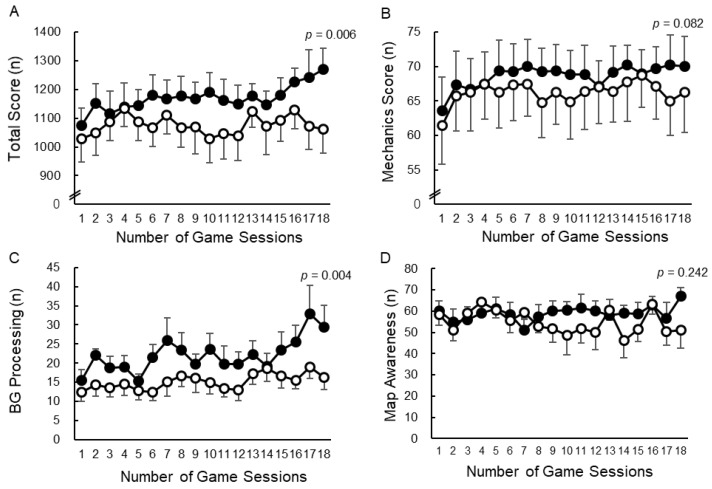
Effects of GABA intake on game scores during esports play. (**A**) Total score and (**B**) mechanics, (**C**) background (BG) processing, and (**D**) map awareness game scores are shown. Closed circles (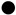) indicate the GABA group, while open circles (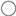) indicate the placebo group. The results indicate the mean ± standard error (SE). Significant differences were examined using a mixed model, with treatment and number of sessions as fixed variables and subject as a random effect (*n* = 8).

Game scores after the consumption of the test supplement were evaluated using a mixed-model analysis (Figure 4). The total score was significantly increased in the GABA group (1170.66, 95% CI: 1131.10, 1210.21) compared to the placebo group (1075.16, 95% CI: 1035.61, 1114.72) (F(1,6) = 16.90, *p* = 0.006) (Figure 4A). Mechanics tended to increase in the GABA group (68.99, 95% CI: 66.49, 71.49) compared to the placebo group (65.92, 95% CI: 63.42, 68.42) (F(1,6) = 4.37, *p* = 0.082) (Figure 4B). Background processing significantly increased in the GABA group (21.95, 95% CI: 19.42, 24.48) compared to the placebo group (15.26, 95% CI: 12.73, 17.79) (F(1,6) = 20.31, *p* = 0.004) (Figure 4C). In terms of map awareness, no significant difference was observed between the GABA group (58.71, 95% CI: 54.04, 63.38) and the placebo group (55.15, 95% CI: 50.49, 59.82) (F(1,6) = 1.683, *p* = 0.242) (Figure 4D).

## 4. Discussion

The aim of this study was to investigate the effects of GABA intake on psychological state and game scores during esports gameplay in eight healthy adult males who play esports games on a daily basis.

The results of the comparison of the POMS 2^®^ short version analysis showed that the GABA group significantly improved subjective CB compared to the placebo group. The CB questionnaire consisted of the following items: confused, unable to concentrate, forgetful, bewildered, and uncertain about things. This suggests that GABA intake improves the subjective state of confusion during a complex task in which the left and right hands instantly perform different processes based on the information obtained from a screen.

Additionally, the GABA group showed significantly improved subjective FI compared to the placebo group. Kanehira et al. [15] also showed that GABA intake reduced subjective fatigue in participants subjected to computational tasks; however, they showed no improvement in subjective confusion. Computational tasks appear to involve serial processing (sequential processing) and do not require sufficient distributed attention to induce a state of CB. In contrast, the MPG used in this study requires parallel processing [21,22], necessitating distributed attention during task execution. This study is the first to show that GABA intake improves subjective confusion in complex tasks that require concurrent and different operations.

The results of game scores in the MPG indicated that the GABA group had significantly increased total scores and background processing compared to the placebo group, with mechanics also showing an increase that approached significance. In the placebo group, game scores tended to decline with repeated trials due to fatigue because this game requires performing multiple actions simultaneously. After each rest point, game scores increased in the placebo group. In contrast, in the GABA group, game scores moderately improved throughout all games. GABA improved subjective CB and FI during the experimental protocol, which involved 18 consecutive rounds of MPG gameplay, and led to a significant increase in total game scores. Mechanics evaluates the ability of a mouse to recognize a target and click it accurately, which is equivalent to attacking enemies or moving in real games. Background processing assesses the ability to perform the parallel task of quickly and accurately operating a keyboard while simultaneously managing the primary task of operating a mouse. This corresponds to a quick-cast operation of skills in real games. GABA intake affected both mechanical and background processing. These findings suggest that GABA intake may be effective for tasks involving multiple operations. In contrast, no significant difference was observed between the GABA and placebo groups in map awareness, which evaluates the ability to check a map used to locate enemies and allies in a real game. This is possibly because the frequency of map awareness operations was lower than that of mechanics and background processing, owing to the task design. Specifically, the input frequency of map awareness (average: 0.13–0.17 times per second), mechanics (average: 1.95 times per second), and background processing (average: 0.77 times per second) are set based on actual measurements. The results of the total score, mechanics, and background processing suggest that GABA intake improves game performance.

MPG is utilized to evaluate game performance [21]. MPG is used as a training game for evaluating player performance in League of Legends (LoL), one of the MOBA genres with the largest number of competitive players worldwide. This game can quantify the mouse and keyboard skills required for gameplay in response to on-screen information and is suitable for evaluating player performance.

MPG was developed by Mobalytics Inc. (Marina Del Rey, CA, USA), an esports analytics company specializing in performance evaluation systems for competitive gaming. Its use in peer-reviewed research—such as by Pluss et al. [21], which examined its reliability and validity as a perceptual-motor skill assessment—supports its relevance in esports research. While the algorithm underlying MPG is proprietary, its core functions have been described in the academic literature, contributing to transparency and enabling reproducibility within the scope of experimental applications.

Although the present study employed a MOBA-type task, which reflects one of the most cognitively and mechanically demanding esports genres, future work could extend these findings by applying similar paradigms to other game types such as first-person shooter (FPS) games, fighting games, or racing games. This would help determine whether the observed effects generalize across varying gameplay mechanics and decision-making contexts, thereby broadening the applicability of MPG-based assessments in esports research.

Of the participants in this study, those who received GABA were administered a 200 mg dose. Recent studies have shown that 28 mg of GABA alleviates subjective stress and fatigue, 100 mg alleviates subjective vitality and vigor, and 200 mg improves cognitive function and subjective vitality [15,16,23]. A GABA intake of 28 or 100 mg has been shown to improve psychological state under mild mental stress conditions in which simple calculation problems are performed. In this study, 200 mg of GABA was administered because an esports game is a highly stressful test in which three tasks are performed simultaneously with different movements of the left and right hands. 

Based on previous studies on GABA, a one-week washout period was implemented. According to Junfeng Li et al. (2015), GABA is rapidly absorbed (T_max_: 0.5–1 h) and has a half-life of approximately 5 h [13]. In pharmacokinetic studies, it is generally recommended that the washout period be at least five times the drug’s half-life [24]. Therefore, the one-week washout period used in this study was considered sufficient to minimize any potential carryover effects.

A hypothesized mechanism, based on previous research, is that the effects of GABA intake on psychological state are possibly mediated through the parasympathetic nervous system. Prior studies have reported that orally ingested GABA appears in the plasma within approximately 30 min [13]. Additionally, several studies have shown that GABA intake increases parasympathetic nervous system activity [25,26] and induces an increase in alpha brain waves, which are associated with relaxed mental states [17,18]. Enhanced parasympathetic activity indicates a moderate state of relaxation. Therefore, GABA intake activates the parasympathetic nervous system, reduces mental stress, and decreases salivary cortisol levels [15]. Moreover, individuals with dominant parasympathetic activity have demonstrated shorter reaction times and better performance in attention tasks, indicating enhanced cognitive functioning [27]. Drawing on prior research, one possible explanation is that this may lead to a moderately relaxed state, which, in turn, could improve psychological status and potentially enhance game performance. Furthermore, recent findings suggest that GABA may influence the brain via the vagus nerve [28]. Vagal-mediated responses are thought to occur more rapidly than those relying on systemic absorption into the bloodstream.

In light of this, the present study adopted an exploratory behavioral design, initiating the performance task approximately one minute after ingestion. This timing was based on prior evidence suggesting early physiological and neurocognitive responses following GABA intake. Although the present study did not directly assess oral bioavailability or related biomarkers, the study design aimed to capture possible short-term effects through behavioral outcomes. Future research could build on these findings by incorporating biochemical measures to clarify the absorption dynamics of GABA and further substantiate the proposed mechanisms.

Previous studies have reported that certain nutritional components enhance esports performance. In trials involving caffeine, the effects were observed 60–110 min after intake [9]. In contrast, the present study implemented a design in which gameplay began 1 min after GABA administration and concluded after 18 rounds in 33 min. Notably, psychological state and game scores improved within 40 min of GABA intake, suggesting an immediate effect. Unlike substances such as caffeine, which crosses the blood–brain barrier to act on adenosine receptors or glucose, the latter of which enhances brain function by serving as a metabolic energy source, GABA may instead exert its effects by modulating mood states through mechanisms involving either vagus nerve signaling or activation of the parasympathetic nervous system, rather than directly influencing arousal or energy metabolism. While certain supplements, such as caffeine, are known to enhance cognitive performance, their excessive intake may cause undesirable side effects, including anxiety, insomnia, and elevated heart rate [29], which warrants careful consideration regarding their use. In contrast, GABA has been reported to be well tolerated in doses up to 2 g per day without major adverse effects [13], suggesting a relatively safe profile as a performance-supporting supplement. These findings suggest that GABA contributes to esports performance in a novel, effective, and safe manner.

This study has several limitations that should be acknowledged. The small sample size (*n* = 8), comprising young male university students with moderate esports experience, limits statistical power and the generalizability of the findings (Table S2). Future research should employ larger samples with more diverse demographics, including professional players, to enhance external validity and confirm these results.

## 5. Conclusions

This study demonstrates that GABA intake effectively reduces subjective confusion and fatigue during esports gameplay, resulting in significantly improved game scores. Thus, GABA intake may serve as a potential strategy to enhance both the mental state and performance of esports players. Although this study specifically examined esports, the results may also be relevant to tasks requiring simultaneous multitasking at work or studying.

## Data Availability

The data presented in this study are available on request from the corresponding author due to ethical reasons.

## Supplementary Materials

The following supporting information can be downloaded at https://www.mdpi.com/article/10.3390/nu17111870/s1: Dataset S1: Phase1.0_dataset (anonymized participant data); Table S1. Analysis of carryover and period effects; Table S2. Results of statistical power analysis.
