# Supplementary material for: γ-Aminobutyric Acid Intake Improves Psychological State and Performance in Esports: A Randomized, Placebo-Controlled, Double-Blind Crossover Study
Source: Nutrients. 2025 May 30;17(11):1870. doi: 10.3390/nu17111870 (PMC12157149; doi:10.3390/nu17111870)
Supplement: Supplementary file 1 [file nutrients-17-01870-s001.zip › Supplementary Materials v4.docx]

**Table S1.** Analysis of carryover and period effects.

|  | **Carryover Effects** | **Period Effect** |
| --- | --- | --- |
| POMS2 short CB | 0.757 | 0.954 |
| POMS2 short FI | 0.731 | 0.722 |
| Total Score | 0.339 | 0.852 |
| Mechanics | 0.226 | 0.116 |
| BG processing | 0.459 | 0.581 |
| Map awareness | 0.342 | 0.327 |

**Table S2.** Results of statistical power analysis.

|  | **Detective Power (1-β)** |
| --- | --- |
| POMS2 short CB | 0.33 |
| POMS2 short FI | 0.44 |
| Total Score | 0.25 |
| Mechanics | 0.07 |
| BG processing | 0.71 |
| Map awareness | 0.14 |
