# Supplementary material for: γ-Aminobutyric Acid Intake Improves Psychological State and Performance in Esports: A Randomized, Placebo-Controlled, Double-Blind Crossover Study
Source: Nutrients. 2025 May 30;17(11):1870. doi: 10.3390/nu17111870 (PMC12157149; doi:10.3390/nu17111870)
Supplement: Supplementary file 1 [file nutrients-17-01870-s001.zip › Supplementary Materials v4.pdf]

Table S1. Analysis of carryover and period effects.

	Carryover Effects	Period Effect
POMS2 short CB	0.757	0.954
POMS2 short FI	0.731	0.722
Total Score	0.339	0.852
Mechanics	0.226	0.116
BG processing	0.459	0.581
Map awareness	0.342	0.327

Table S2. Results of statistical power analysis.

	**Detective Power (1-β)**
POMS2 short CB	0.33
POMS2 short FI	0.44
Total Score	0.25
Mechanics	0.07
BG processing	0.71
Map awareness	0.14
